# Implications of miR cluster 143/145 as universal anti-oncomiRs and their dysregulation during tumorigenesis

**DOI:** 10.1186/s12935-015-0247-4

**Published:** 2015-09-29

**Authors:** Ani V. Das, Radhakrishna M. Pillai

**Affiliations:** Cancer Research Program-9, Rajiv Gandhi Centre for Biotechnology, Thycaud.P.O., Thiruvananthapuram-14, Kerala India

**Keywords:** MicroRNAs, miR cluster 143/145, Anti-oncomiR, Multidrug resistance, Tumorigenesis

## Abstract

Tumorigenesis is a multistep process, de-regulated due to the imbalance of oncogenes as well as anti-oncogenes, resulting in disruption of tissue homeostasis. In many cases the effect of oncogenes and anti-oncogenes are mediated by various other molecules such as microRNAs. microRNAs are small non-coding RNAs established to post-transcriptionally regulate more than half of the protein coding genes. miR cluster 143/145 is one such cancer-related microRNA cluster which is down-regulated in most of the cancers and is able to hinder tumorigenesis by targeting tumor-associated genes. The fact that they could sensitize drug-resistant cancer cells by targeting multidrug resistant genes makes them potent tools to target cancer cells. Their low levels precede events which lead to cancer progression and therefore could be considered also as biomarkers to stage the disease. Interestingly, evidence suggests the existence of several in vivo mechanisms by which this cluster is differentially regulated at the molecular level to keep their levels low in cancer. In this review, we summarize the roles of miR cluster 143/145 in cancer, their potential prognostic applications and also their regulation during tumorigenesis.

## Background

Cancer is a complex condition arising from the accumulation of several genetic alterations privileged to deregulated cell division. Extensive research to unveil the molecular mechanisms of tumorigenesis led to the characterization of a large number of genes as oncogenes and anti-oncogenes. It was thought that alterations in these molecules are the reasons leading to cancerous growth until the involvement of microRNAs (miRNAs) was exposed. miRNAs are endogenous small (about 22 bp in length); non-coding, regulatory RNAs present in a wide variety of organisms and are located in the intronic or non-intronic regions of protein-coding genes transcribed either along with the genes or independently. miRNAs are transcribed as long primary RNAs by RNA polymerase II [1] which then undergo two steps of processing: first in the nucleus by the RNase III-type protein Drosha [2] and DGCR8 [3, 4] generating pre-miRNAs and second by Dicer, after exported to the cytoplasm by Exportin-5 or Exportin-1 [5], to produce mature miRNAs. Mature miRNAs then act as negative regulators of gene functions by becoming a part of the RNA-induced silencing complex (RISC) [1] and target their downstream mRNAs by base-pairing to their complementary sequences mostly at the 3′UTR region which results in the degradation of target mRNAs and/or inhibition of translation, thereby decreasing the specific gene expression [6, 7]. Most miRNAs are evolutionarily conserved and display diverse temporal and tissue-specific expression pattern [8–10]. A single miRNA can target and regulate more than hundreds of mRNAs, and one mRNA can be targeted by multiple miRNAs [11–13]. miRNAs contribute to a different level of molecular regulation, thus being involved in various roles in cellular and developmental functions, such as dorso-ventral axis and temporal pattern formation [14, 15], cell death and cell proliferation [16, 17], neuronal differentiation [18], stem cell proliferation and maintenance [19, 20] and also, in embryonic development [21].

Recently, miRNAs have gained tremendous attention in the field of cancer research. Altered miRNA expression can lead to cellular de-differentiation, oncogenesis, cancer metastasis and tumor invasion [22]. miRNA de-regulation is considered as a common hallmark of cancer [23], scoring miRNAs as important diagnostic and therapeutic targets. Calin et al. reported for the first time that two miRNAs, miR-15 and miR-16, were involved in the development of chronic lymphocytic leukemia [24] followed by He et al. who demonstrated that expression of miR-17-92 cluster could enhance c-Myc-induced tumorigenesis marking this cluster to be the first potential non-coding oncogene, referred to as oncomiR-1 [25]. Likewise, *let*-*7* family of miRNAs was reported to regulate expression of a proto-oncogene, the RAS protein [26, 27], and hence were coined as anti-oncomiRs. Later, many miRNAs have been reported to have roles in oncogenesis and miR cluster 143/145 is one among them having anti-oncogenic effects in many cancers which are being discussed in this review in detail.

## miR cluster 143/145

**Fig. 1:**
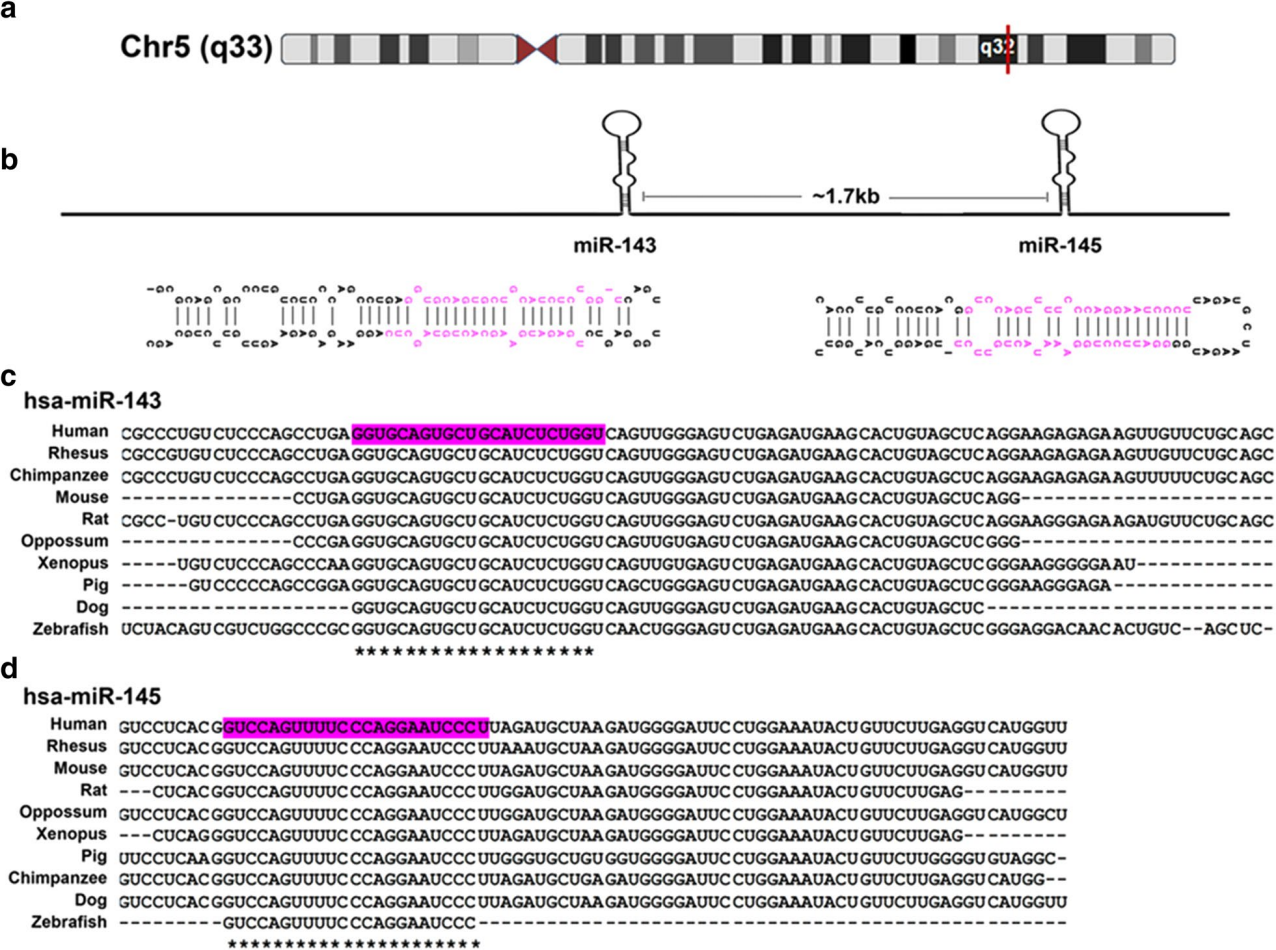
miR cluster 143/145 is evolutionarily conserved across species. **a** Schematic of chromosomal location of miR cluster 143/145 (adapted from UCSC genome browser. **b** Schematic representation of structures of miR cluster 143/145 primary transcripts and their location. Multi-species alignment of sequences of miR-143 (**c**) and miR-145 (**d**) (courtesy to Clustal W Omega)

miR cluster 143/145 comprises of two miRNAs, miR-143 and miR-145, that have significant roles in various cellular functions and are co-expressed in a variety of cell types and tissues [28]. These miRNAs are transcribed from a putative cluster on chromosome 5 in human (5q33) and chromosome 18 in mouse (18qE1), and are conserved across species (Fig. 1). miR-143 is separated from miR-145 by ~1.7 kb sequence [28]. Since they are in the same cluster and suggested to be transcribed together, it was speculated that they could be involved in similar functions. However, independent involvement of these miRNAs is also reported in many cellular processes. Both miR-143 and miR-145 are expressed in normal tissues in significant levels, with highest expression in colon and lowest in liver and brain [28]. The expression of these miRNAs was considerably high in prostate, cervix, stomach, uterus and small intestine and low in kidney, placenta, testis, spleen and skeletal muscle [28]. This cluster is found enriched in embryonic stem cells which differentiate into cardiac progenitors [29] suggesting an involvement in cardiac morphogenesis. They play a very important role in the fate specification of vascular smooth muscle cells since they target a number of transcription factors to inhibit proliferation in order to promote differentiation [29].

## miR cluster 143/145 in cancers

miR-143 and miR-145 are now regarded as tumor suppressors since they target a number of genes involved in the tumorigenesis (Table 1), and their deregulation has been reported as one of the early events in cancer development [30, 31]. Both miR-143 and miR-145 are commonly seen down-regulated in a wide variety of cancer cell lines and tumors of the hematopoietic system, breast, lung, colon, prostate, the gastrointestinal system, ovary, cervix, head and neck, bladder [23, 32, 33], endocrine cancers such as thyroid, pituitary and gonads [34], germ-cell tumors (GCTs) [35], gallbladder cancer [36, 37], renal cell carcinoma [38–40], osteosarcoma [41, 42], and neuroblastoma [43, 44]. The reduced miR-145 expression in prostate cancer (PCa) samples correlated with higher Gleason score, advanced stage, tumor size, higher prostate-specific antigen (PSA) and significantly shorter disease-free survival (DFS) for the PCa patients [45] and also associated with poor prognosis, lymph node metastasis and advanced stage in cervical cancers [46]. Low levels of miR-143 was negatively correlated with tumor size and lymph node metastasis in breast cancer while that of miR-145 was associated with dysplastic nodules, Hepatitis C virus-infection and metastasis in hepatocellular carcinoma (HCC) [47–49]. The observation that ALDH^+ve^/CD44^+ve^ cancer stem cells showed low levels of miR-145 reinforced its importance as an effective approach to target the stem cell population in cancer [50]. Similar observations were made in case of glioma as well, where the decreased levels of miR cluster 143/145 were positively correlated with poor prognosis and negatively correlated with ABCG2, suggesting that miR-145 could efficiently target stem cell-like populations and reduce the migration and invasion of such cells [51]. miR cluster 143/145 has also been shown to play crucial role in the pathogenesis of B cell malignancies. It is suggested that miRNAs on chromosome 5q have an important role in leukemia and many of the miRNAs on the Chr:5q including miR-145 have been found to be deregulated in leukemia.

**Table 1:** Validated targets for miR cluster 143/145 with their cancer-related function

Target genes	Type of cancer	Cancer-related function	References
*miR-143*			
KRAS	CRC, PCa, PaCa	Proliferation	[109]
MACC1	CRC	Metastasis	[110]
TLR2	CRC	Proliferation, invasion	[109]
DNMT3A	CRC, BrCa, leukemia	Proliferation	[111–113]
Akt	CRC, BC, HCC, glioma	Apoptosis, proliferation	[114–117]
ERK5	CRC, PCa, BC, B-cell malignancy	Proliferation	[115, 117–119]
Bcl2	CRC, BrCa, OC, BC	Apoptosis	[60, 92, 115, 120]
Survivin	BrCa	Apoptosis	[89]
ARHGEF1	PaCa	Metastasis	[92]
ARHGEF2	PaCa	Proliferation, invasion	[121]
FNDC3B	HCC	Metastasis	[121]
Cox2	BC, GC	Metastasis	[117, 131]
MMP-13	OC, lung	Invasion	[122, 123]
Lmk1	Lung	Metastasis	[124]
*miR-145*			
Cateninδ-1	CRC	Proliferation, invasion	[125]
DFF45	CRC	Apoptosis	[126]
VEGF	CRC, BrCa, OC, thyroid, GB	Angiogenesis	[127–130]
c-Myc	CRC, PCa, EOC, BrCa, PaCa, ESCC, lung, glioma, RCC	Proliferation, invasion, apoptosis	[58, 131–134]
PAK4	CRC	Proliferation, invasion	[135]
IRS1	CRC, HCC, BC	Proliferation	[49]
IRS2	CRC	Proliferation	[84]
YES	CRC	Proliferation	[136]
STAT1	CRC	Proliferation	[136]
Fascin-1	CRC, BrCa, BC, ESCC, glioma	Invasion	[106, 137–140]
SWAP70	PCa	Invasion	[141]
ERG	PCa	Proliferation, invasion, apoptosis, angiogenesis	[142]
CD44	PCa, lung	CSC	[132, 134, 143]
Oct4	PCa, lung, GCT	CSC	[132]
KLF4	PCa, GCT	CSC	[144]
CDK4	Lung	Proliferation	[145]
CDK6	EOC, OSCC	Proliferation	[146]
p70S6K1	CRC, EOC	Proliferation	[146]
Muc-1	EOC, BrCa	Invasion	[146, 147]
RTKN	BrCa	Proliferation, invasion	[148, 149]
JAM-A	BrCa	Proliferation	[150]
ERα	BrCa	Proliferation	[83]
RREB1	PaCa	Proliferation, apoptosis	[74]
HDAC2	HCC	Transcriptional regulation	[103]
Ets1	GC	Migration, apoptosis, angiogenesis	[151]
N-cadherin	GC	Invasion	[152]
E-cadherin	Thyroid	Invasion	[130]
EGFR	Lung, glioma	Proliferation	[153, 154]
NUDT1	BC, lung	Proliferation	[153]
ROCK1	OC, glioma	Proliferation, migration	[155, 156]
FLT1	OC	Proliferation	[157]
PAK1	BC	Proliferation, metastasis	[158]
CBFβ	BC	Apoptosis	[159]
PPP3CA	BC	Apoptosis	[159]
CLINT1	BC	Apoptosis	[159]
SOCS7	BC	Apoptosis	[160]
Ilk	BC	Cell division/proliferation	[161]
ANGPT2	RCC	Angiogenesis	[40]
NEDD9	Glioma, RCC	Proliferation, metastasis	[40, 98]
MMP-11	RCC	Metastasis	[162]
CTGF	Glioma	Proliferation, invasion, metastasis, angiogenesis	[104]
ADAM17	Glioma, RCC, HNSCC	Proliferation, invasion, metastasis, angiogenesis	[96]
ADAM22	Glioma	Metastasis, drug resistance	[106]
Abcg2	Glioma	Drug resistance, CSC	[51]
ADD3	Glioma	Proliferation, invasion	[70]
Sox9	Glioma, HNSCC	Proliferation, CSC	[70, 72]
Sox2	Glioma, GCT	Proliferation, CSC	[97, 163]
Nanog	Glioblastoma	CSC	[97]
PLAUR	Glioblastoma	Metastasis	[106]
SPOCK3	Glioma	Invasion	[106]
SLC7A5	Glioma	Proliferation, metastasis	[106]
AKT3	Thyroid	Metastasis	[205]
*miR-143 and miR-145*			
KLF5	CRC	Proliferation	[61]
Myo6	PCa	Migration, apoptosis	[164]
GOLM1	PCa	Metastasis	[165]
CD133	PCa	CSC	[132]
IGFIR	CRC, HCC, BC	Proliferation	[114, 166, 167]
MDM2	BrCa, HNSCC	Apoptosis	[65]
PAI-1	BC	Migration, metastasis	[168]
HK2	BrCa, HNSCC, OC, RCC, glioma	Tumor initiation and maintenance	[42, 86, 169–172]
N-ras	BrCa, glioma	Cell division, proliferation, apoptosis	[117, 128, 173, 174]
ERBB3	BrCa	Drug resistance	[175]

*CRC* colorectal cancer, *PCa* prostate cancer, *PaCa* pancreatic cancer, *BrCa* breast cancer, *GC* gastric cancer, *HCC* hepatocellular carcinoma, *OC* osteosarcoma, *RCC* renal cell carcinoma, *BC* bladder cancer, *HNSCC* head and neck squamous cell carcinoma, *GCT* germ cell tumor, *OSCC* oral squamous cell carcinoma, *GB* gall bladder, *EOC* epithelial ovarian cancer, *ESCC* esophageal squamous cell carcinoma

Very interestingly, a significant correlation of miR cluster 143/145 expression with environment-mediated cancer development was found in the case of lung cancer. Lung cancer is associated with environmental carcinogens such as cigarette, air pollution, and heavy metals. Chronic exposure of chromium [Cr(VI)], one such heavy metal widely used in industries, to non-tumorigenic human lung epithelial BEAS-2B cells resulted in the repression of miR-143 which in turn led to the malignant transformation, suggesting that the effect of environmental carcinogens could be mediated by miRNAs. Similarly, in malignant pleural mesothelioma (MPM), an aggressive cancer associated with long-term exposure to asbestos, miR cluster 143/145 was found to be significantly down-regulated, suggesting that these miRNAs may serve as suitable biomarkers for distinguishing MPM from non-cancerous pleural tissues [52, 53]. Smoking, another cause for lung cancer, has also a negative effect on miR cluster 143/145 expression. miR-145 was one of the mostly down-regulated miRNAs in cigarette smoke-exposed lungs of rodents [54]. Whether this effect could predict malignant transformation, needs further investigation.

## miR cluster 143/145 and multidrug resistance

Multidrug resistance is a phenomenon where cells develop resistance to a range of cytotoxic agents by effluxing them out with the help of transporter proteins. Though these transporter proteins, otherwise known as multidrug resistant proteins (MDR), are crucial for cell survival, their high expression in cancer cells has been a significant obstacle to successful chemotherapy. miR cluster 143/145 has been known to regulate MDRs in various cancers. miR-145 is reported to inhibit MDR1 in intestinal cells [55] and ABCG2 in glioma cells as we all as in corneal cells [56]. The reduction in miR-145 expression could be related to drug resistance potential of many cancer cells [57]. Reduced levels of miR-145 caused increase in the levels of Sp1 and CDK6 thereby reducing the levels of Pgp and pRb, thus suggesting a possible the reason for increased chemoresistance in ovarian cancer cells [58]. Ectopic expression of miR-145 increased the sensitivity of cells to various drugs such as paclitaxel and adriamycin in cervical cancer [58], as well as vemurafenib [59], 5-FU, irinotecan and oxaliplatin [60, 61] in colorectal cancer. In glioblastoma cells, miR-145 could sensitize the cells to temozolomide as well as to irradiation [62]. Likewise, adenoviral mediated over-expression of miR-145 (Ad-miR-145) in breast cancer cells increased the sensitivity to 5-FU in vitro and in vivo, suggesting that a combination of miR-145 with drugs like 5-FU could be a possible option to target breast cancer cells [63]. The combined introduction of miR-143 and miR-145 in gastric cancer cell line, MKN-1 cells resulted in a higher sensitivity to 5-fluorouracil (5-FU) [64]. Also, inhibition of MDM2 either by miR-143 or miR-145 sensitized HN30 cells to cisplatin, suggesting that this cluster is able to reduce the chemoresistance in HNSCC cells too [65]. Curcumin, a proven chemosensitizing agent in cancer cells, has shown to activate miR-145 expression in HNSCC cells [50] suggesting that the chemosensitizing action of certain agents could be mediated by miRNAs. Also, miR-143 could induce chemosensitivity towards docetaxel in prostate cancer [66] and miR-143-mediated inhibition of Lmk1 enhanced the sensitivity of the NSCLC cells to chemotherapy [67]. Role of miR cluster 143/145 in sensitizing cancer cells to drugs is thus an area of significance in understanding therapeutic interventions in cancer.

## Regulation of miR cluster 143/145 in cancers

The fact that in most of the cancers miR cluster 143/145 was found to be de-regulated points towards the existence of specific mechanisms that regulate their expression in cancer cells. When analyzed at the genetic level, loss of heterogeneity in the miR cluster 143/145 loci was detected in a number of ovarian carcinoma samples [68]. Li et al. found 12 Single nucleotide polymorphisms (SNPs) in the promoter region of miR-143/145 that could attribute to the etiology of colorectal tumors [69]. Increased methylation at CpG islands on miR-145 promoter could be one reason for the reduced levels which is observed in many cancers [70]. Also, HDAC has shown to up-regulate miR cluster 143/145 in Burkitt’s lymphoma cells [71]. On the other hand, Peroxisome proliferator-activated receptor γ (PPARγ) could activate miR-145 by binding to PPARγ-responsive element present on the upstream sequence of miR-145 promoter [72].

Evidence suggests that many oncogenic as well as anti-oncogenic factors mediate their effects through activation or inactivation of miR cluster 143/145 (Table 2). For example, EGFR signals negatively regulate miR cluster 143/145 thereby removing the suppression on many positive regulators of tumorigenesis [73–76]. Ras-responsive element binding factor (RREB1), which is downstream to KRAS-MAPK signaling, was found to down- regulate miR 143/145 cluster expression [75]. Through RREB1, KRAS independently and/or together with members of MAPK and PI3K, has been shown to repress miR 143/145 cluster in pancreatic cancer cells [74]. On the other hand, TGFβ, secreted by cells in scirrhous type of gastric cancer, could activate miR-143 expression in the neighboring stromal fibroblasts thus inducing their proliferation through activation of collagen type III [77]. Up-regulation of miR-145 mediated via p65NFkB was also observed in response to Resistin, an adipocyte-derived cytokine, thereby stimulating insulin resistance in HepG2 cells [78]. Similarly, FoxO, a transcription factor which acts down stream to insulin and insulin-like growth factor receptor pathways, suppress c-Myc in RCC cells by up-regulating miR-145 along with Mxi1-SRα [79]. BRCA-1, a suppressor of breast cancer, has also proved to be an activator of miR-145 through directly interacting with DROSHA microprocessor complex [80]. Also, some of the antitumor effects shown by p53 are mediated through miR-145 since the abrogation of miR-145 in p53-over-expressed cells reversed the inhibition of p53 on migration, invasion, EMT and stemness of PC3 cells [81, 82] and also could be a reason for suppression of cell growth in vitro and in vivo in HNSCC [50] and breast cancer cells [83]. The fact that activation of p53 pathway results in elevation of expression levels of both miR-143 and miR-145 [84, 85] confirms involvement of this cluster in tumor suppression. An interesting observation is that miR-155 has been shown to negatively regulate miR-143 via targeting C/EBPb, a transcriptional activator of miR-143 in breast cancer cells [86].

**Table 2:** List of molecules that regulate miR cluster 143/145 in cancer

Regulatory molecules	Cancer type	Expression of these regulators in cancer	miRNA	References
*Positive regulators*				
FoxO	RCC	Low	miR-145	[79]
p53	CRC, BrCa, PCa, HNSCC, Cervical	Low	miR-143, miR-145	[50, 83–85]
TGF-β1	GC	High	miR-143	[77]
BRCA1	BrCa	Low	miR-145	[80]
C/EBPβ	BrCa	Low	miR-143	[86]
PPARγ	CRC	Low	miR-145	[72]
*Negative regulators*				
HPV-E6	HPV-induced cervical	High	miR-145	[88]
RREB1	CRC	High	miR-143, miR-145	[74]
KRAS	CRC, PaCa	High	miR-143, miR-145	[74]
EGFR	CRC	High	miR-143, miR-145	[73–76]
ERα	GC	High	miR-143, miR-145	[89, 90]
17-β-estradiol	BrCa	High	miR-143	[92, 93]
Estrogen	BrCa	High	miR-143, miR-145	[94]
FSH	EOC, cervical	High	miR-143	[176]
Adam17	RCC	High	miR-145	[96]
Sox2	Glioma, GCTs	High	miR-143, miR-145	[97]
Limk1	NSCLC	High	miR-143	[67]
DDX6	GC	High	miR-143, miR-145	[177]

*CRC* colorectal cancer, *PCa* prostate cancer, *PaCa* pancreatic cancer, *BrCa* breast cancer, *GC* gastric cancer, *RCC* renal cell carcinoma, *HNSCC* head and neck squamous cell carcinoma, *EOC* epithelial ovarian cancer, *GCT* germ cell tumor, *NSCLC* non-small cell lung carcinoma

There are various other factors including hormones which have been proved to regulate the expression of miR cluster 143/145 in various cancers. For instance, follicle stimulating hormone (FSH) has been shown to negatively regulate the expression of miR-143 in cervical cells [87]. Cortisol also could reduce miR-145 expression by suppressing p53 and this may also be mediated by HPV-E6 expression in HPV-infected cervical cells [88]. It has been reported that ERα inhibits processing of several microRNAs, including miR-145 and miR-143 [89, 90]. ER-α36, whose up-regulation is positively correlated with lymph node metastasis was able to repress miR-143 levels in gastric cancer cells [91]. Likewise 17-β-estradiol (E2)-mediated inhibition of miR-143 could be attributed to the increased proliferation in many cancers [92, 93]. While miR-143 is inhibited by E2, miR-145 has been reported to be regulated by estrogen [94], mediated through the ER binding region within upstream regulatory region of miR cluster 143/145 [95].

Few targets of miR cluster 143/145 were reported to repress the expression of this cluster, establishing a double negative feedback loop. For example, ADAM17, a proven target of miR-145, could negatively regulate miR-145 expression in RCC cells [96]. In gliobloastoma, both miR-143 and miR-145 have been identified as direct targets of Sox2 whose interaction led to the down-regulation of miR cluster 143/145 which revealed a double negative feedback loop [97]. Another target of miR-145, NEDD9, suppressed miR-145 expression in glioma [98]. Similarly, miR-143 target Limk1 could negatively regulate miR-143 expression in NSCLC cells [67]. Another negative feedback regulation was found in the case of HPV^+ve^ cervical cancers. HPV negatively regulated miR-145 in a differentiation-dependent manner [99]. Together, these data suggest that de-regulation of miR cluster 143/145 could add to the incidence as well as progression of cancer.

## miR cluster 143/145 and cancer therapy

miR cluster 143/145 has been demonstrated to be anti-oncogenic in several cancers, which emphasizes the use of this cluster in a therapeutical approach to treat cancers. As of now, miR cluster 143/145 has been shown to impart their anti-oncogenic effects at various levels including inhibition of proliferation, down-regulation of oncogenes, blocking cell invasion and migration, inducing apoptosis and promoting differentiation. A number of in vivo experiments by various groups have proved the ability of miR cluster 143/145 to intervene oncogenic properties of the cancer cells. Polyethylenimine (PEI)-mediated delivery of miR-145, either systemically or locally, to the tumors in mouse xenograft models led to decreased tumor growth, increased apoptosis and inhibition of targets such as c-myc and ERK5 in colon cancer cells [100]. Preliminary experiments with synthetic mimics of these miRNAs suggested that the stable form of such synthetic mimics could be used as therapeutic tool for treating cancers [101, 102]. miRNAs in their original form are easily degradable. To overcome this, chemically modified analogs could be used. As an effort it was found that addition of an aromatic compound type (3′-benzene-pyridine; BP) to the 3′-overhang region of the RNA-strand enhanced the stability of miRNAs. Such stabilized miR-143 (miR-143BP) was able show tumor suppressive effects on CRC cells [102]. In another approach, subcutaneous injection of miR-145 transfected-Hep3B cells into athymic nude mice showed an overall reduction in tumor growth rate and average volume of the tumors [103].

According to a recent report, mesenchymal stem cells (MSCs) could be used as vehicles to deliver miRNAs. In osteosarcoma and gliomas, MSCs were used for effective delivery of miR-145 since MSCs have migrating ability and can easily migrate into the tumors [104]. Introduction of exosome enveloped-miR-143, derived from synthetic miR-143-transfected MSC-conditioned media, significantly reduced the invasion and migration of OC cells and this particular technique can be used for efficient delivery of miRNAs into target cells [105]. Another group that tried retroviral-mediated delivery of miR cluster 143/145 in PaCa cells observed a reduced anchorage-independent growth, though they were unable to find a reduction in the total proliferation rate. Interestingly, miR cluster 143/145 expressing MiaPaCa2 and Panc-1 cells were also unable to form tumors in immune-compromised mice [74]. Adenoviral-mediated ectopic expression of miR-145 using Ad5CMV.Rz.HSVtk.miR145 exerted an enhanced antitumor effect in U87MG/U373MG glioma cells suggesting a possible combination therapy using the hTERT.Rz.HSVtk gene together with miR-145 [106].

Formulation of a proper delivery system is essential for miRNAs to be used in therapies. Pramanik et al. [107] took another step and delivered vectors expressing miR-143/145 conjugated with liposomal nanoparticles in mice. Briefly, nanovectors containing miR143/145 delivered through tail vein of MiaPaCa-2-xenografted mice induced a significant reduction in the tumor size. Upon delivery of miR-143/145, levels of KRAS-2 and RREB1, two known targets of this cluster, were significantly inhibited in the xenografts. Bacteriophage capsid mediated delivery system for miRNAs has also become promising effort in this aspect. miRNAs encapsulated with virus-like particles (VLPs) of bacteriophage MS2 after conjugating with modified HIV-1 Tat47-57 peptide with sulfoSMPB has been reported as an efficient vehicle for delivering miRNAs and could be used to deliver miR cluster 143/145 efficiently to the tumor cells also [108]. Together, these findings are encouraging in the possible therapeutic use of this particular cluster as anti-cancer agents.

## Conclusions

**Fig. 2:**
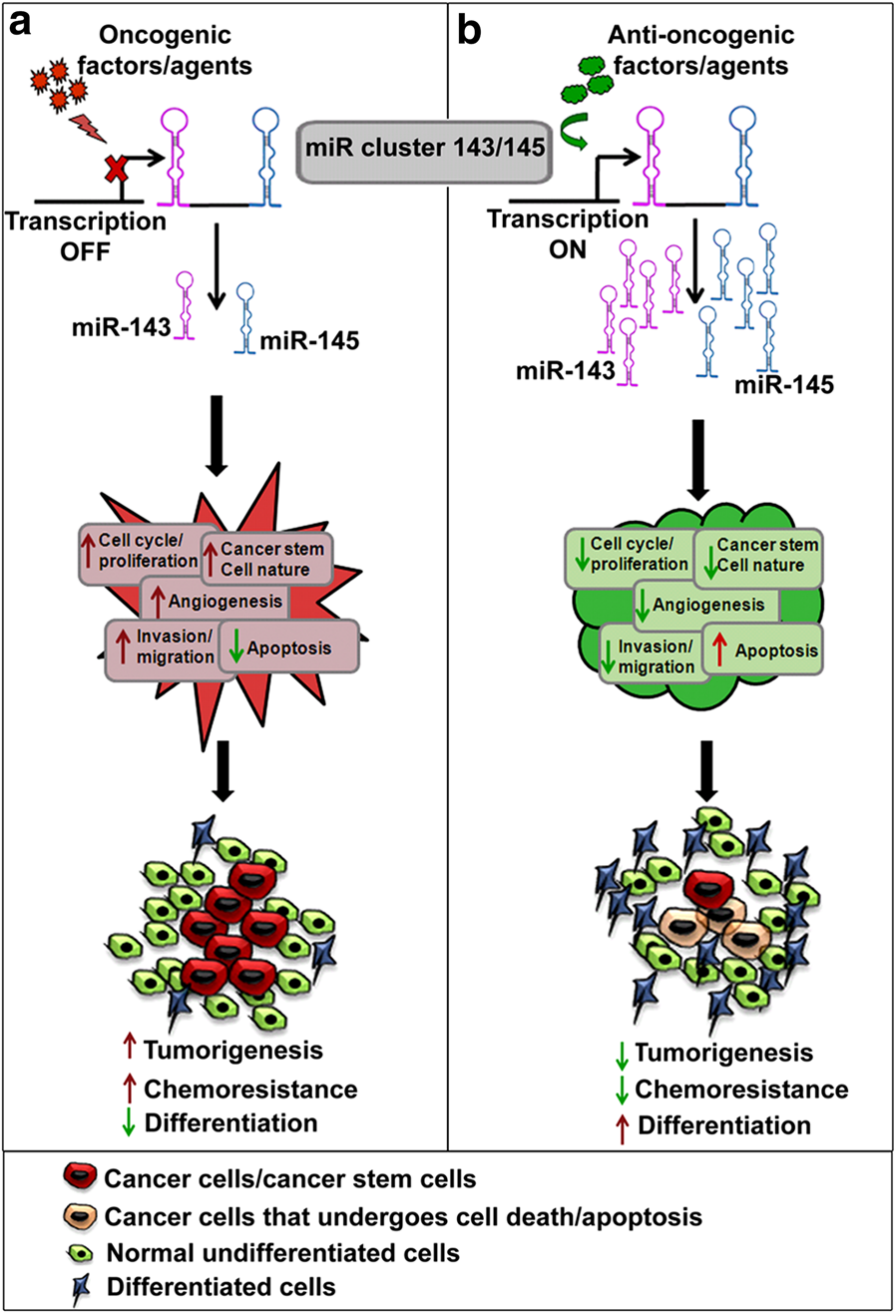
Schematic representing the involvement of miR cluster 143/145 in tumorigenesis. **a** Represents that some oncogenic signals can reduce the transcription of miR cluster 143/145 which in turn increase the tumorigenic events resulting in cancer. **b** On the other hand, anti-oncogenic signals can up-regulate this cluster which eventually can reduce tumorigenesis

The role of miR cluster 143/145 in cancer is of significance since both miR-143 and miR-145 have been shown to suppress tumorigenesis by targeting various genes that play significant roles during the development of cancer. Though these miRNAs have some common targets, they do have specific targets too, thus they act in concert or independently to impart the functions. On the other hand, miR cluster 143/145 is regulated, either positively or negatively, by various factors in cancer cells (Fig. 2). Evidence suggests that many oncogenes repress miR cluster 143/145 in order to impart their oncogenic effects in those cells, whereas anti-oncogenic factors, including transcription factors and drugs, elicit their effects through up-regulation of miR cluster 143/145. The mechanism of their regulation is different is different cell types. Both these miRNAs are supposed to be under the control of a common promoter, and are found to follow a similar pattern of expression in most of the cases. Since miR-145 has a specific upstream regulatory element of ~1.5 kb length, it could be possible that this miRNA is regulated independent of miR-143. This might be a possible reason for the disparity in the expression pattern of miR-145 and miR-143 in some cell types. However, the reason behind their differential regulation in different cell types is still unclear. More importantly, recent findings suggest that both the miRNAs play an important role in sensitizing cancer cells to various drugs which could be useful for formulating better combination therapy options for cancer. Moreover, expression levels of these miRNAs are most of the time reflected in the serum, suggesting their use as biomarkers for understanding prognosis of the disease. There have been few promising steps taken at the laboratory level to deliver these miRNAs efficiently to tumor sites and have to be investigated further. Taking all these evidences into consideration, miR cluster 143/145 can be regarded as ideal candidates for therapeutic interventions for cancers.

## Abbreviations

DGCR8: DiGeorge syndrome critical region 8
RISC: RNA-induced silencing complex
ABCG2: ATP-binding cassette, subfamily G
MDR1: multidrug resistance protein 1
CDK6: cyclin-dependant kinase 6
Pgp: *P*-gycoprotein
pRb: retinoblastoma protein
5-FU: 5-fluorouracil
MDM2: mouse double minute homolog 2
HNSCC: head and neck squamous cell carcinoma
NSCLC: non-small cell lung carcinoma
RCC: renal cell carcinoma
CRC: colorectal cancer
PaCa: pancreatic cancer
HDAC: histone deacetylase
EGFR: epidermal growth factor receptor
KRAS: v-Ki-ras2 Kirsten rat sarcoma viral oncogene homolog
MAPK: mitogen-activated protein kinase
TGFβ: transforming growth factor beta
PI3K: phosphoinositide-3-kinase
BRCA-1: breast cancer-1, early onset
FoxO: forkhead protein O
EMT: epithelial-to-mesenchymal transition
ERα: estrogen receptor α
HPV: human papilloma virus
ADAM17: ADAM metallopeptidase domain 17
Sox2: SRY_box 2
NEDD9: neural precursor cell expressed, developmentally down-regulated 9
Limk1: LIM domain kinase 1
ERK5: extracellular signal-regulated kinase 5
